# Construction of axially chiral molecules enabled by photoinduced enantioselective reactions

**DOI:** 10.1039/d4sc03766a

**Published:** 2024-07-25

**Authors:** Zhaofei Zhang, Lei Dai

**Affiliations:** a Chongqing Key Laboratory of Natural Product Synthesis and Drug Research, Chemical Biology Research Center, School of Pharmaceutical Sciences, Chongqing University Chongqing 401331 China dailei@cqu.edu.cn; b Department of Chemistry, Purdue University West Lafayette Indiana 47907 USA

## Abstract

Axially chiral molecular scaffolds are widely found in pharmaceutical molecules, functionalized materials, and chiral ligands. The synthesis of these compounds has garnered considerable interest from both academia and industry. The construction of such molecules, enabled by transition metal catalysis and organocatalysis under thermodynamic conditions, has been extensively studied and well-reviewed. In recent years, photoinduced enantioselective reactions have emerged as powerful methods for the catalytic construction of axial chirality. In this review, we provide an overview of various synthetic strategies for the photoinduced construction of axial chirality, with a specific focus on reaction design and catalytic mechanisms. Additionally, we discuss the limitations of current methods and highlight future directions in this field.

## Abstract 2

An overview of the recent advances in the photoinduced construction of axially chiral compounds was presented.

## Graphical abstract

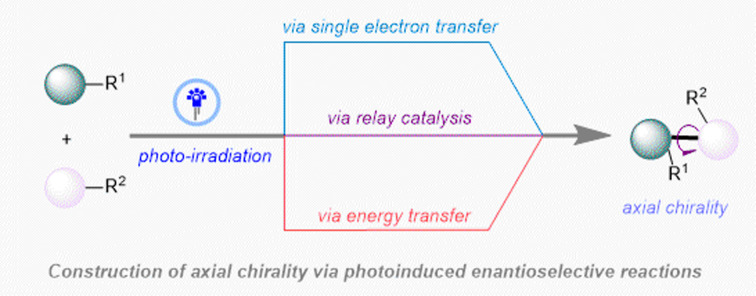

## 1. Introduction

**Scheme 1:**
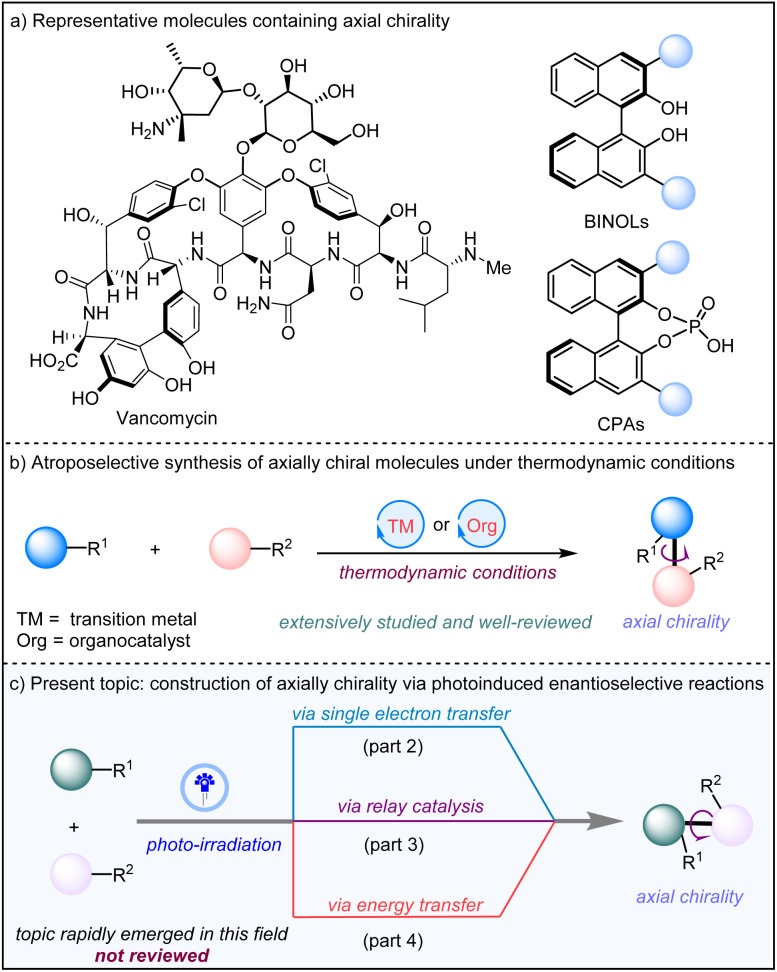
Strategies for the photoinduced construction of axial chirality.

Axially chiral molecular scaffolds widely exist in bioactive molecules and pharmaceutical agents,^1^ as well as privileged chiral organocatalysts and ligands in asymmetric catalysis (Scheme 1a).^2^ Consequently, atroposelective construction of these axially chiral compounds is of growing interest to chemists in both academia and industry.^3^ The construction of axial chirality enabled by transition metal catalysis and organocatalysis *via* a two-electron pathway has been extensively studied, with many impressive achievements reported (Scheme 1b).^3^ The significant applications of axially chiral compounds notwithstanding, expanding substrate scope and developing sustainable and cost-effective methods are still in high demand.

Visible light photocatalysis^4^ has gained immense attention and witnessed great development in recent years, as it can activate substrates *via* single electron transfer (SET) or energy transfer (EnT)^5^ to generate key radical intermediates leading to a variety of transformations, which are difficult to obtain under standard thermodynamic conditions. Since the pioneering report by the MacMillan group^6^ for the construction of central chirality, many strategies for enantioselective visible-light photocatalysis have been designed by incorporating transition metal catalysis^7^ and organocatalysis (Scheme 1b).^8^ Despite the significant success of photoinduced construction of central chirality, the construction of axial chirality by photocatalysis has become an intriguing topic of tremendous potential. However, there remain some daunting tasks, such as incorporating unactivated substrates into the reaction scope, developing general methods for radical generation,^9^ and efficiently controlling the stereochemistry of radical-mediated reactions under photocatalysis.

In this review, we will present an overview of the recent advances of photoinduced construction of axially chiral compounds. Based on the activation modes of substrates by photocatalysis, three strategies will be discussed (Scheme 1c). The first strategy relies on the generation of radical intermediates through photoredox mediated SET, and the following stereoselective reactions of the generated radical intermediates lead to axially chiral compounds. Second, relay photocatalysis and asymmetric transformation are described. Third, photoinduced *via* energy transfer is also involved, where a key intermediate is formed to facilitate the deracemization reaction. Lastly, we will also provide a discussion on the existing limitations and possible future directions in this field.

## 2. Construction of axial chirality *via* photoinduced single electron transfer

Enantioselective metallaphotocatalysis^10^ has become a powerful strategy to access chiral compounds over the past decade. Since the first report by the Molander group,^10*c*^ asymmetric metallaphotoredox catalysis to access central chirality has been well studied. Nevertheless, its application for the construction of axial chirality has been far less developed.

**Scheme 2:**
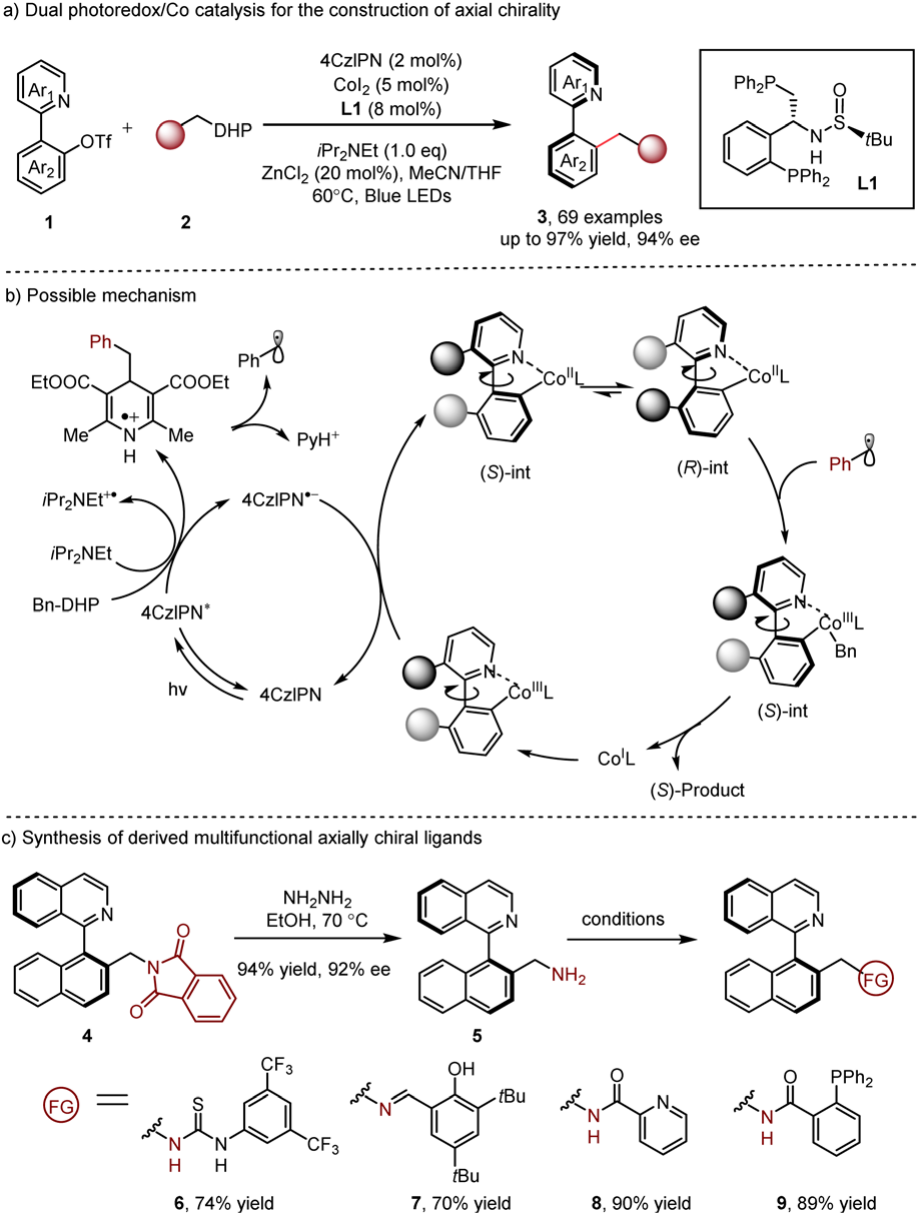
Dual photoredox/Co catalysis for the construction of axial chirality.

In 2022, the Xiao and Lu group^11^ reported a dual photoredox/cobalt^12^ catalyzed dynamic kinetic resolution (DKR) of transformation of racemic heterobiaryls 1 with 1,4-dihydropyridine (DHP) reagent 2, producing axially chiral heterobiaryls 3 in excellent yields with excellent enantioselectivities (Scheme 2). Wei-Phos L1 (ref. 13) was screened and it could afford the best enantioselectivity. In their reaction design, the chiral cobalt catalyst coordinated to the nitrogen atom of the substrate, leading to the generation of a configurationally labile complex. The following photogenerated radical trap of (*S*)-int and the subsequent reductive elimination delivered the final product. Notably, the scope of radical precursors was successfully extended to alkyl chlorides,^14^ demonstrating the great generality and practicality of this method. Moreover, several derived multifunctional axially chiral ligands 6–9 could be accessed through simple procedures, presenting high potential for future applications (Scheme 2c). This enantioselective dual photoredox/Co catalysis offers a new valuable alternative for the synthesis of axially chiral compounds accommodating flexible and various substitution patterns.

**Scheme 3:**
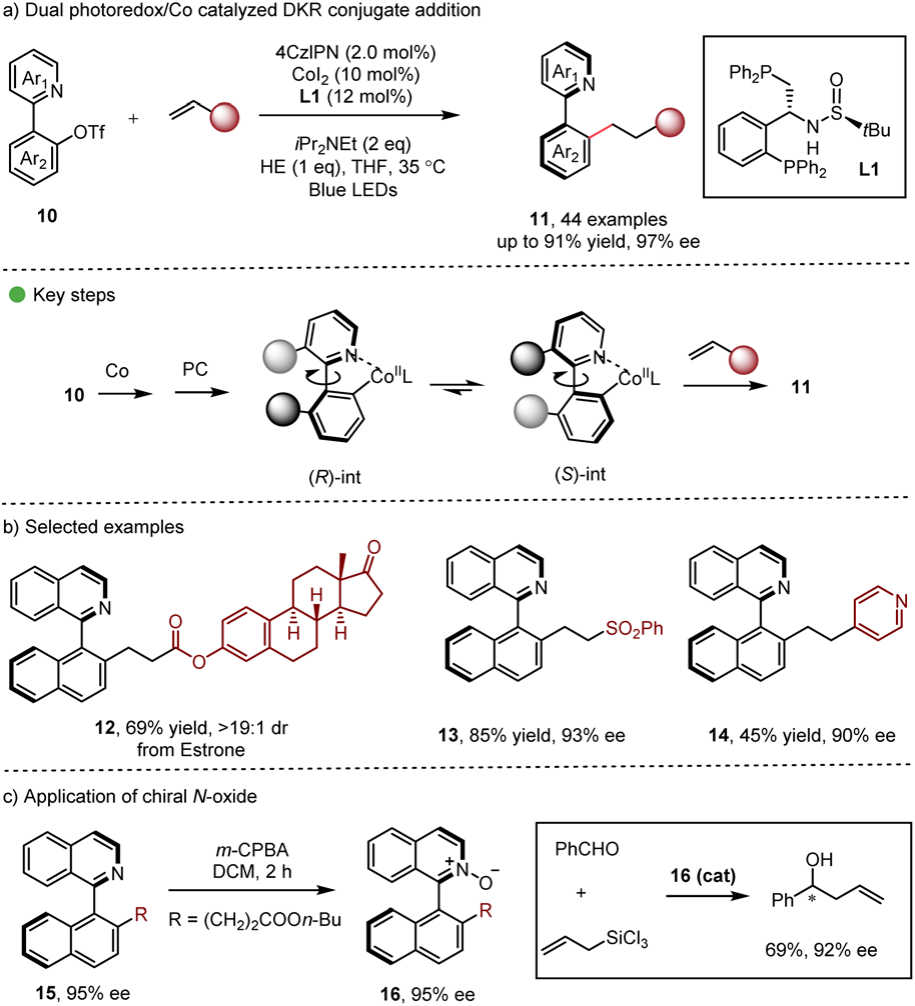
Dual photoredox/Co catalyzed DKR conjugate addition.

Continuing with their research interest, Xiao, Gao and co-workers employed synergistic photoredox-cobalt catalysis in DKR conjugative addition (Scheme 3) for the synthesis of enantioenriched heterobiaryls 11 in good to excellent yields.^15^ Various functionalities, such as ester, cyano, amido, carbonyl, heteroaryl, sulfonyl and phosphonyl groups could be introduced into the axially chiral products, which holds significant potential for the development of axially chiral ligands. Interestingly, the derived *N*-oxide 16 showed excellent enantiocontrol in the asymmetric allylation reaction of aldehydes (Scheme 3c). It should be noted that reductive cobalt catalysis was also viable with 30 eq. of Zn as the reductant, demonstrating certain practicality of the dual catalytic method.

**Scheme 4:**
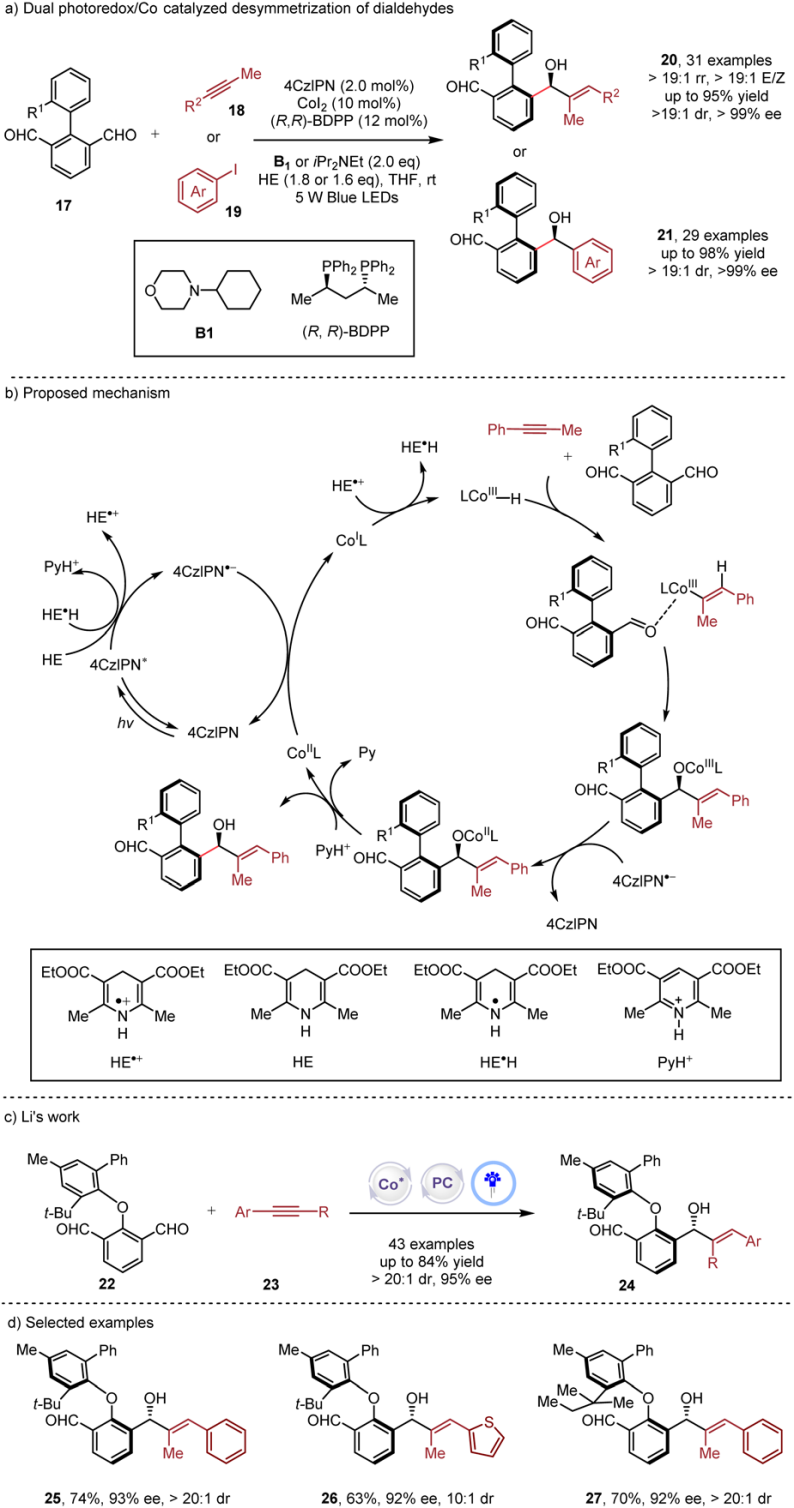
Dual photoredox/Co catalyzed desymmetrization of dialdehydes.

Simultaneous construction of both axial chirality and central chirality^16^*via* enantioselective metallaphotocatalysis was disclosed by the Xiao and Cheng group (Scheme 4a) in the desymmetrization of diaryl based dialdehydes 17.^17^ The synergistic use of photoredox and cobalt catalysis showcased high efficiency in the reductive coupling of alkynes 18 or aryl iodines 19, achieving exceptional stereocontrol with a broad range of substrate scope. Additionally, the versatilities of aldehyde and alkyne in the products allowed for multiple derivatizations of the products. Very recently, the Li group (Scheme 4c) employed a similar strategy in the desymmetrization of diaryl ethers 22 with alkynes 23 under metallaphotocatalysis.^18^ While the dual catalysis could afford excellent reactivities and stereocontrol, the reductive cobalt catalysis delivered only a trace amount of product, underscoring the superiority of the dual catalysis.

**Scheme 5:**
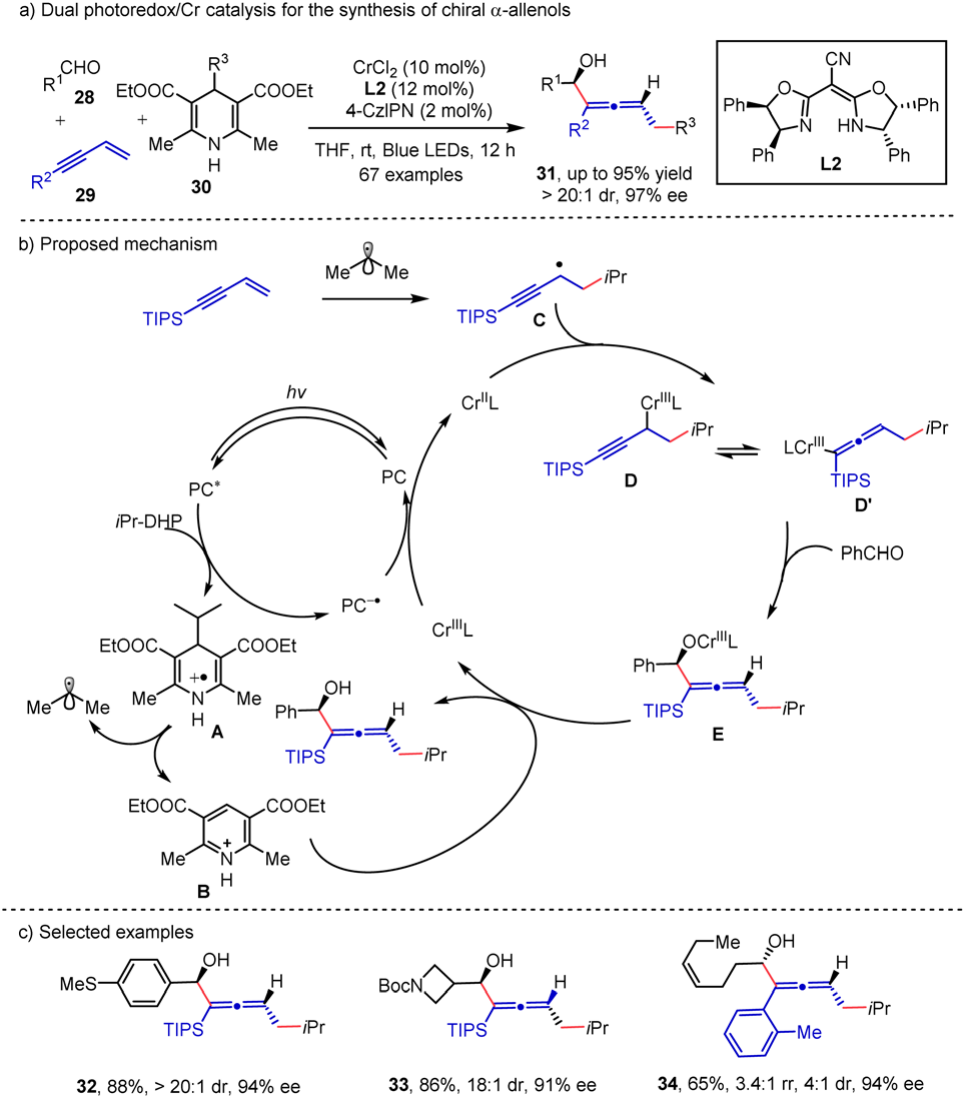
Dual photoredox/Cr catalysis for the synthesis of chiral α-allenols.

In recent years, radical mediated 1,4-difunctionalization of 1,3-enynes^19^ served as an efficient method for the synthesis of chiral allene compounds. With the advances in photocatalysis, numerous methods have been developed for the synthesis of a diverse range of functionalized allenes. Asymmetric synthesis of allene mediated by photocatalysis was disclosed by the Wang group^20^ where aldehydes 28, 1,3-enynes 29 and DHP esters 30 were incorporated in a three-component reaction (Scheme 5). In their proposed mechanism, single electron oxidation of DHP ester by the photocatalyst generates an alkyl radical and pyridinium A. The radical addition to 1,3-enyne and the subsequent trapping by the Cr^II^ catalyst lead to propargyl radical chromium D, which is in equilibrium with the alkenyl chromium intermediate D′. The alkenylation product readily yields the intermediate E, and the dissociation of the Cr–O bond in E by pyridium B delivers the desired product. Finally, the SET reduction of Cr^III^ closes the catalytic cycle. The regioselectivity might be attributed to the use of the steric bulky substitution group (TIPS). Simultaneous control of axial and central chirality is successfully achieved, producing the corresponding chiral α-allenols 31 in good to excellent yields with excellent diastereoselectivities and enantioselectivities (up to 95% yield, >20 : 1 dr & 97% ee).

**Scheme 6:**
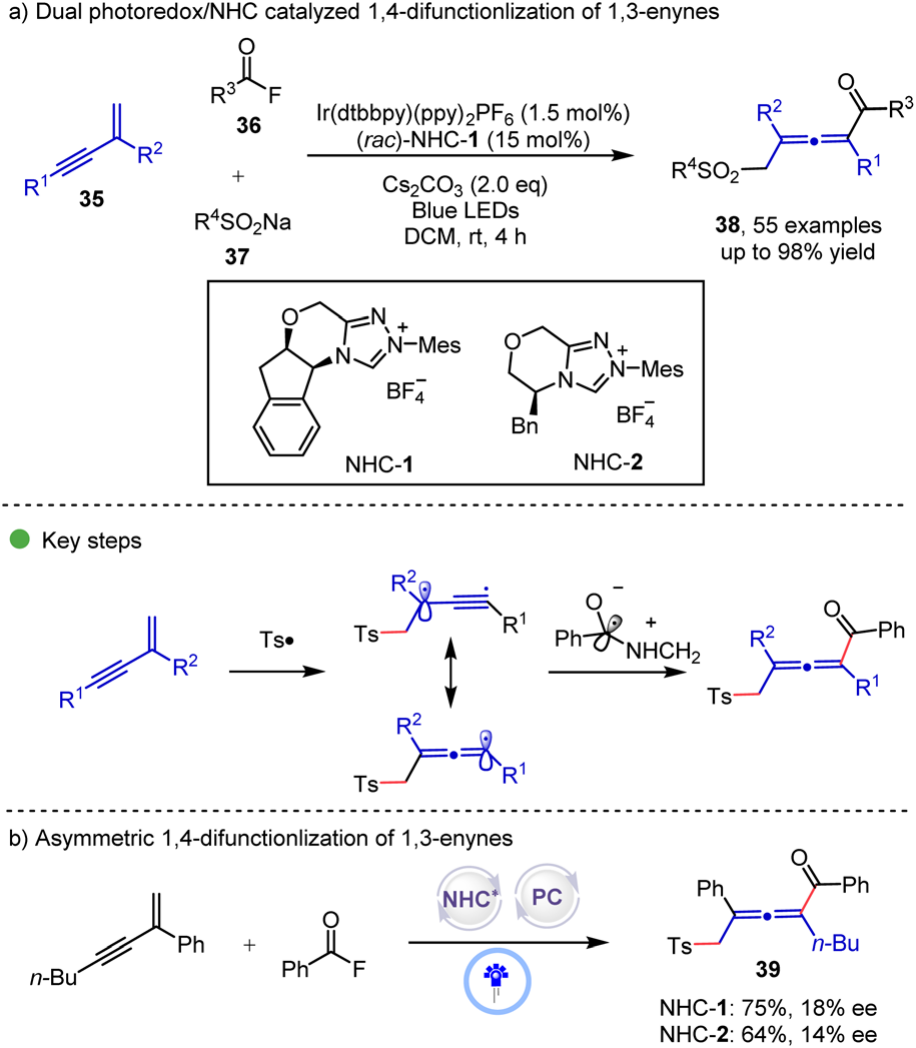
Dual photoredox/NHC catalyzed 1,4-difunctionalization of 1,3-enynes.

Radical mediated N-heterocycle carbene (NHC) catalysis opens a new avenue for organic synthesis.^21^ In 2022, the Zhang and Zheng group^22^ reported a dual photoredox/NHC catalyzed 1,4-sulfonylacylation of 1,3-enynes 35 for the synthesis of tetrasubstituted allenyl ketones 38 (Scheme 6). In the proposed mechanism, a photogenerated sulfonyl radical generated from sulfinate undergoes radical addition to 1,3-enynes to form an allenyl radical, which then undergoes radical–radical coupling with the photogenerated ketyl radical from the combination of acyl fluoride and the NHC catalyst to afford the final product. In the optimization of reaction conditions, the low concentration is critical for achieving high yield. The asymmetric version of this reaction was also investigated, however, only poor enantioselectivities were obtained when NHC-1 and NHC-2 were used.

**Scheme 7:**
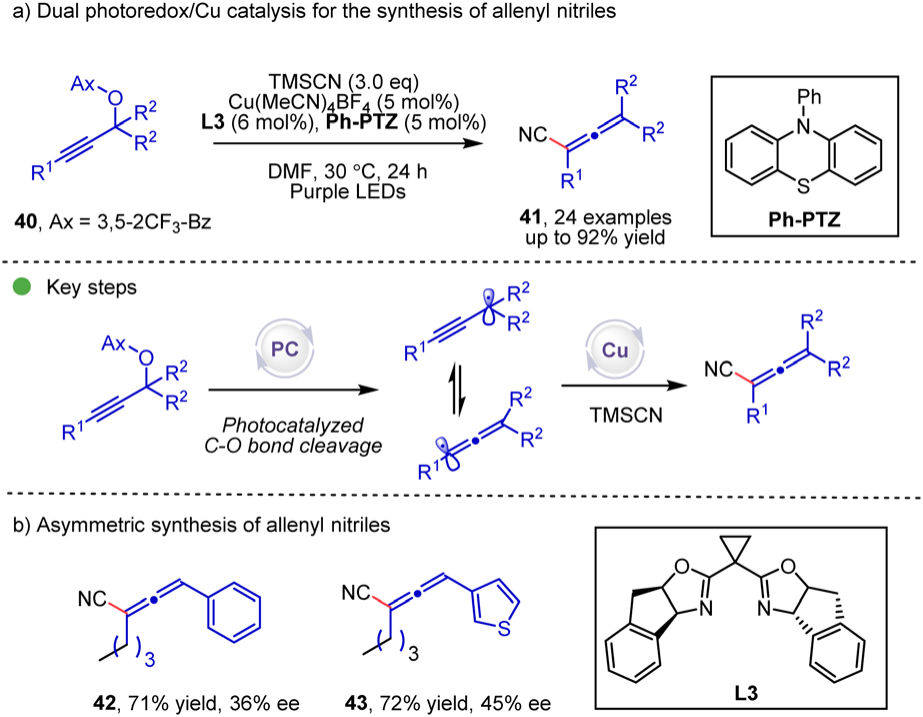
Dual photoredox/Cu catalysis for the synthesis of allenyl nitriles.

The propargyl radical, which could isomerize into an allenyl radical, holds great potential for the synthesis of allene compounds. The dual photoredox/copper (Scheme 7) catalyzed transformation of propargyl carbonate 40 to allenyl compounds was discovered by the Xiao and Lu group.^23^ During the reaction, the allenyl radical could be generated by the photocatalytic C–O bond cleavage and the subsequent isomerization, and then this allenyl radical participates in the copper catalytic cycle to afford the allenyl nitrile products. In the substrate scope, when the R group is an alkyl group, chiral allenes are formed, albeit with moderate enantioselectivities.

**Scheme 8:**
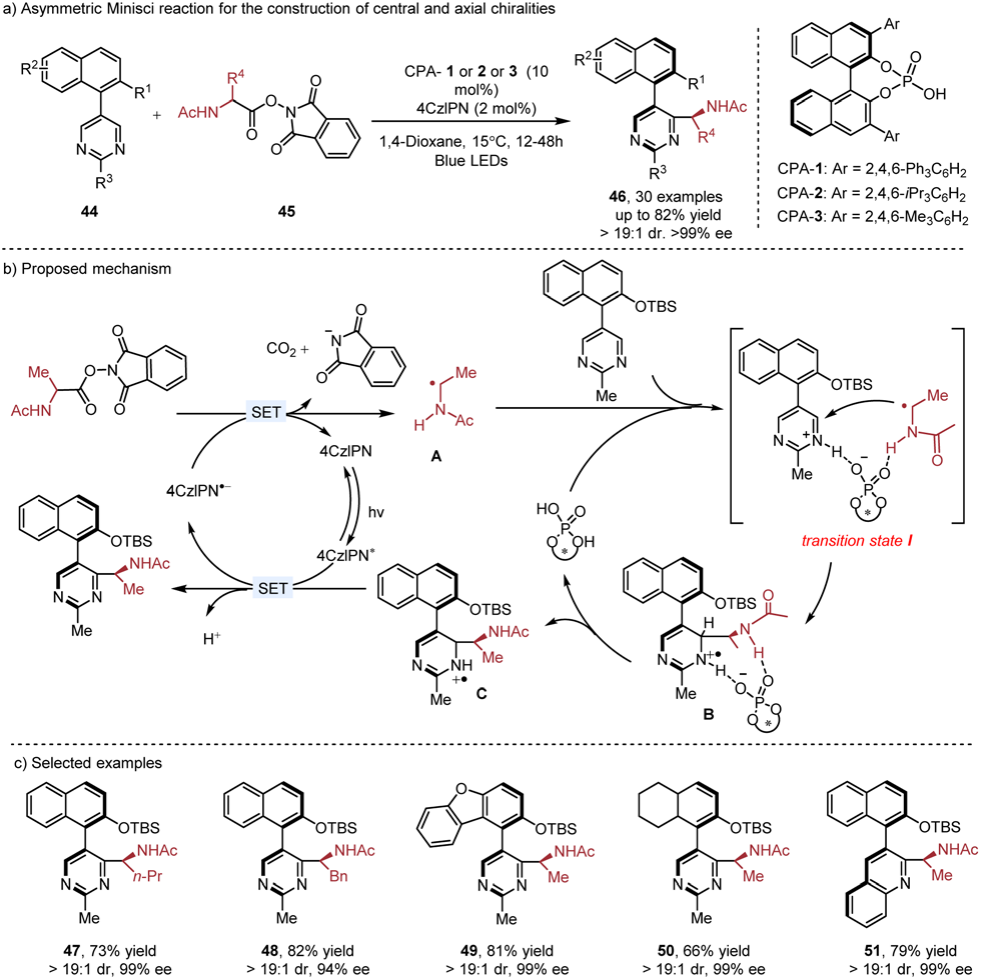
Asymmetric Minisci reaction for the construction of central and axial chirality.

Chiral phosphoric acid (CPA) catalyzed enantioselective Minisci reaction has emerged as an efficient method to provide chiral functionalized *N*-heteroarenes with central chirality.^8*c*^ Catalytic construction of axial chirality^24^ using this strategy was introduced by the Xiao group in 2022 (Scheme 8). When the heterobiaryls 44 were used as the substrates in the Minisci reaction, the axial chirality could be achieved as well as central chirality. In their proposed mechanism, the radical addition of photogenerated radical A to pyrimidines occurs in the presence of CPA through the transition state I. Subsequent deprotonation of radical cation B by the CPA catalyst is the rate-determine step, leading to the radical intermediate C. The following SET oxidation, deprotonation and rearomatization produce the final product. The protecting group (TBS) in the substrate is crucial for achieving excellent diastereoselectivities and it can be easily removed. The resulting chiral amino alcohol ligand shows excellent enantioselectivities in the asymmetric alkylation of aldehydes.

**Scheme 9:**
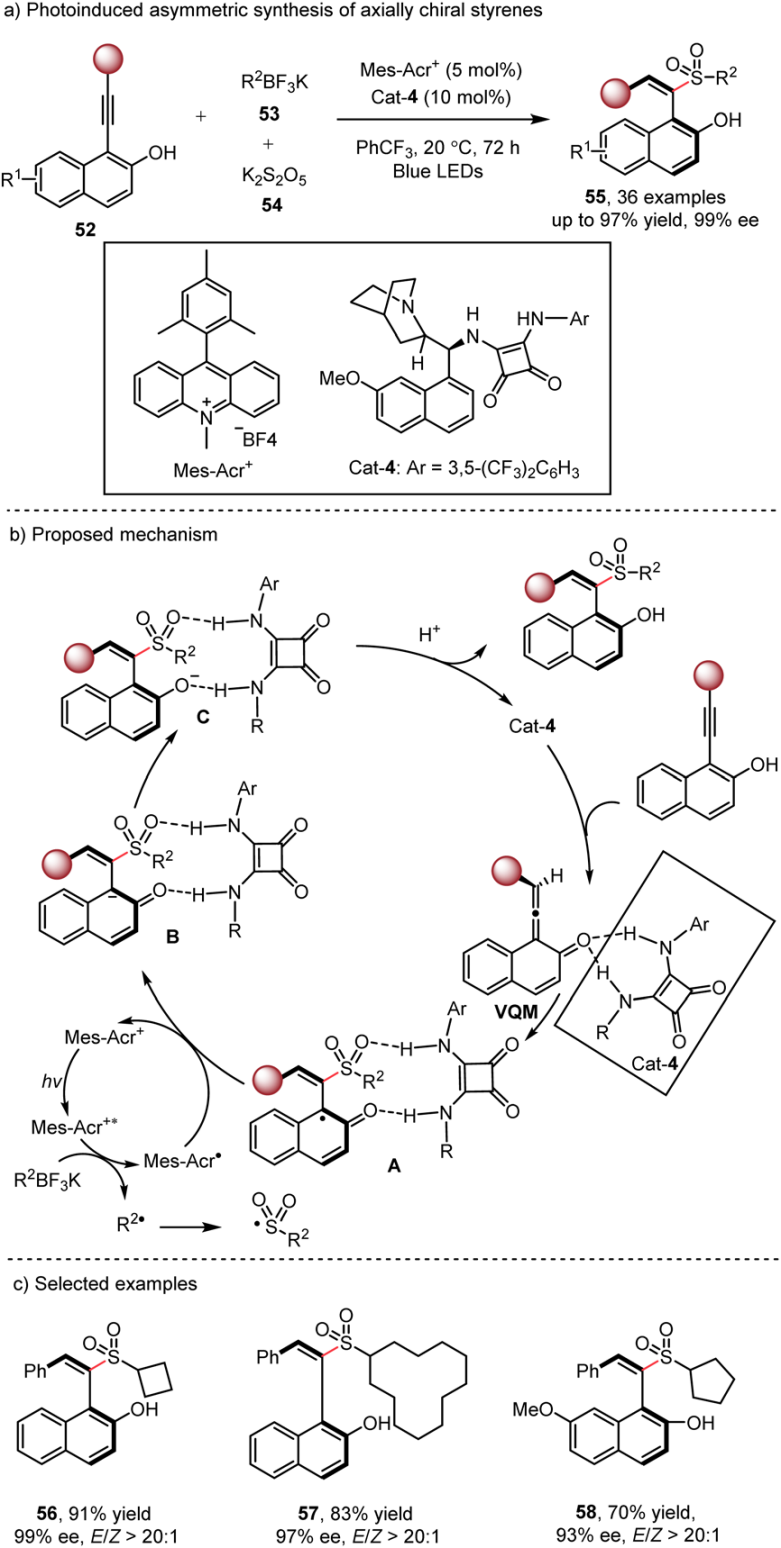
Photoinduced asymmetric synthesis of axially chiral styrenes.

Axially chiral alkenes are widely used as chiral ligands, catalysts and functional materials,^25^ and therefore catalytic asymmetric synthesis of these compounds has gained tremendous attention. In 2017, the Yan group reported an elegant method for the synthesis of axially chiral sulfone-containing styrenes through the organocatalyzed nucleophilic addition of sulfinate salts *via* the vinylidene *o*-quinone methide (VQM) intermediate.^26^ However, this method was limited to aryl sulfinates as the nucleophilic reagents. To expand the scope, the Wu group^27^ developed a dual photoredox/organocatalytic method for the synthesis of a series of chiral alkyl sulfone styrenes 55 (Scheme 9). Upon visible light irradiation, the photogenerated alkyl radical reacts with (DABCO)(SO_2_)_2_ to form a sulfonyl radical. Concurrently, the VQM intermediate is formed *via* prototrophic rearrangement in the presence of the bifunctional organocatalyst. Then the radical addition of the sulfonyl radical to the central carbon of the prochiral VQM intermediate produces intermediate A. Subsequent photocatalyzed SET reduction, tautomerization and protonation yield the final product. The bifunctional organocatalyst exhibits excellent reactivity and stereocontrol for the reaction.

## 3. Construction of axial chirality *via* photoinduced relay catalysis

**Scheme 10:**
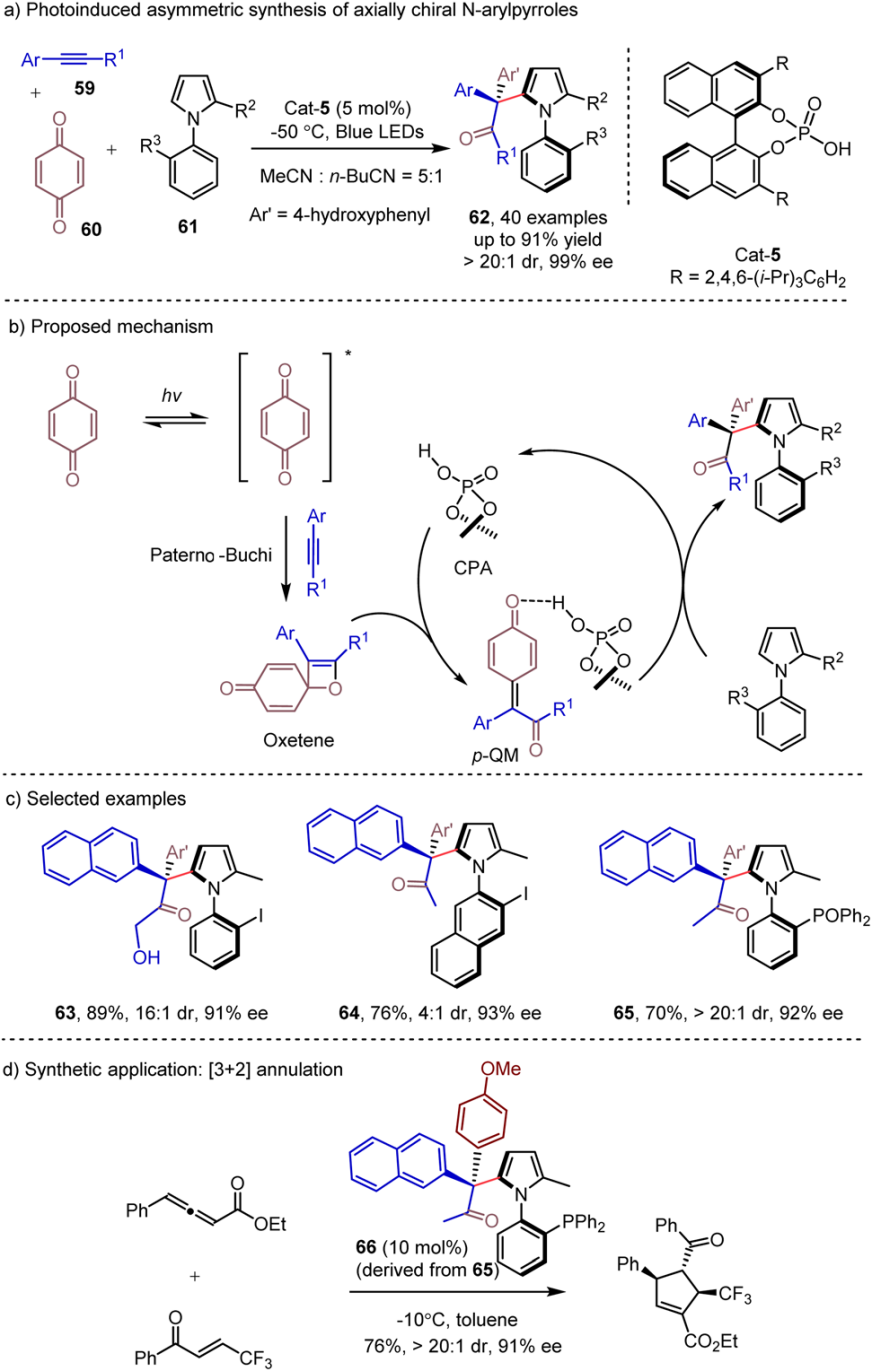
Photoinduced asymmetric synthesis of axially chiral *N*-arylpyrroles.

Photoinduced relay catalysis, relying on the generation of key intermediates *via* photocatalysis, has been established for developing novel reaction modes and enabling the synthesis of structural diverse molecules.^28^ Visible-light-driven [2 + 2] photo-cycloadditions provide an efficient and direct approach to functionalized cyclobutanes,^29^ which could serve as versatile synthons in organic synthesis. In 2022, Lu and Dai^30^ discovered that under visible light irradiation, the [2 + 2] cycloaddition of alkynes and benzoquinones could proceed to give oxetane intermediates. This intermediate could lead to *para*-quinone methide (*p*-QM) in the presence of the CPA catalyst. This key prochiral intermediate could then be readily trapped by external nucleophiles, such as indole, Hantzsch esters or *d*_2_-Hantzsch ester to deliver the corresponding products with high enantioselectivities and high deuterium incorporation. The *N*-arylpyrroles 61 have also been demonstrated as efficient external nucleophiles in the photoinduced transformation,^31^ which could afford enantioenriched axially chiral *N*-arylpyrroles 62 containing a quaternary carbon center and C–N axial chirality (Scheme 10). The ultraviolet-visible spectra revealed that only benzoquinone was photoexcited under visible light irradiation. Employing *p*-QM as the substrate with *N*-arylpyrrole under the same conditions could lead to the product with a similar result, suggesting that the *p*-QM might be formed during the catalytic process. The control experiment showed that CPA and light were crucial for this transformation. Benefiting from the use of readily available alkynes, a broad range of axially chiral *N*-arylpyrroles 62 were synthesized in excellent yields with exclusive regioselectivities and excellent enantioselectivities. Moreover, the derived chiral phosphine ligand 66 was successfully obtained through simple chemical transformations, and it showed excellent enantioselective control in the organocatalyzed [3 + 2] annulation reaction and Pd catalyzed allylic substitution reaction (Scheme 10d).

## 4. Construction of axial chirality *via* photoinduced energy transfer

**Scheme 11:**
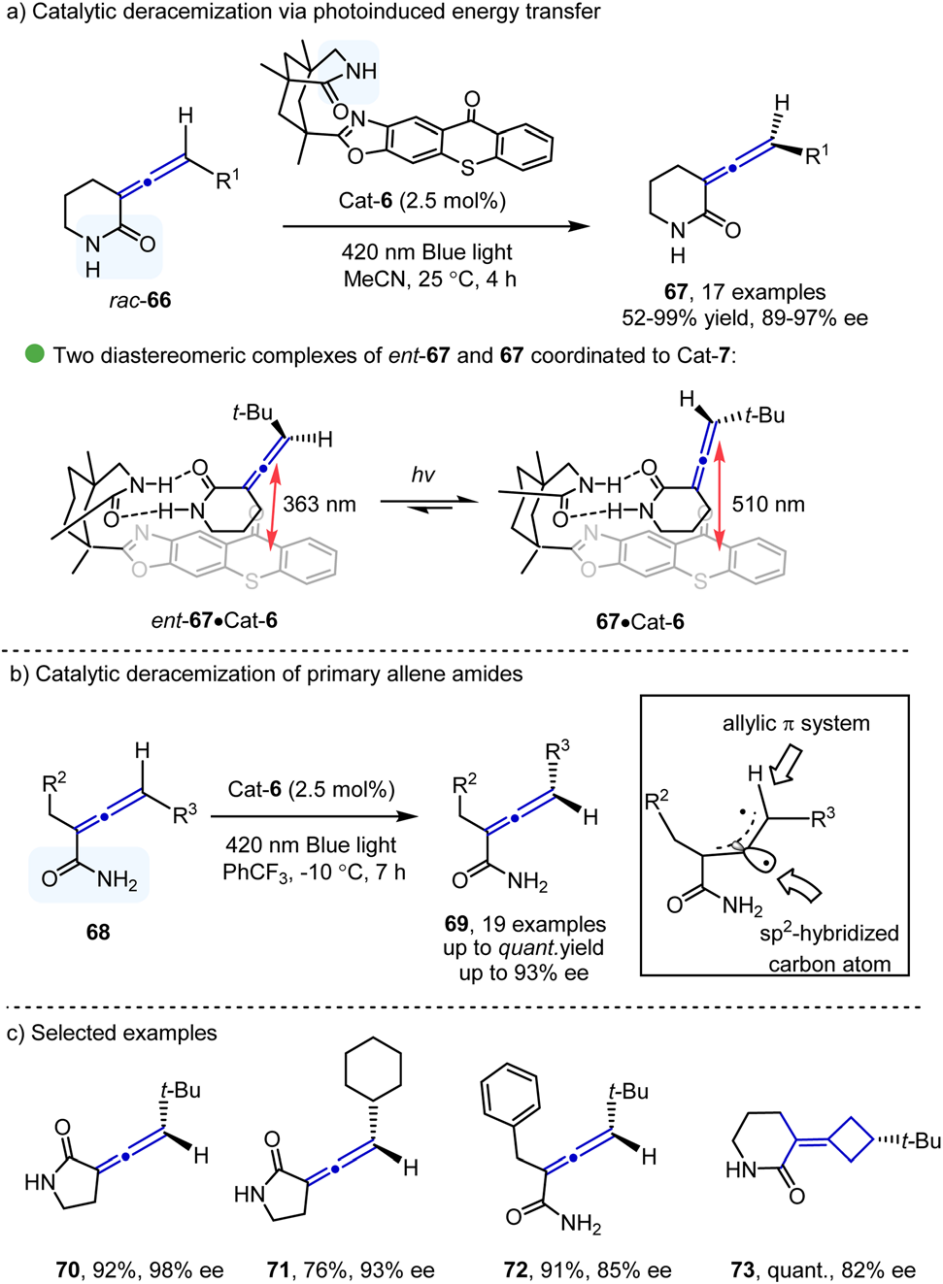
Catalytic deracemization *via* photoinduced energy transfer.

Catalytic deracemization^32^ has emerged as a powerful tool for the asymmetric synthesis of chiral compounds with 100% atom economy. With the development of photocatalysis *via* the energy transfer process,^33^ this research area has seen a renaissance, especially in the synthesis of axially chiral compounds. The pioneering study for photoinduced deracemization of penta-2,3-diene was reported in 1973,^34^ however, poor enantioselectivity (up to 3.4%) was observed. A significant breakthrough in this area came from the Bach group in 2004,^35^ who demonstrated the construction of central chirality. The photoinduced deracemization of the construction of axial chirality was disclosed by the same group in 2018 (Scheme 11a). They reported an elegant deracemization of allenes 66 catalyzed by bifunctional chiral thioxanthones under blue LED irradiation, providing the corresponding axially chiral allenes 67 bearing six-membered lactam in excellent yields with enantioselectivities. DFT calculations showed that the amide moiety in the catalyst could selectively recognize the amide group in the allene, thus differentiating two diastereomeric complexes of the two allene amides (Scheme 11a). In contrast to 67–Cat-6, the *ent*-67–Cat-6 complex has a shorter distance between the thioxanthone photosensitizer and the substrate, which could result in a more rapid triplet energy transfer to convert to the other enantiomer *via* the triplet intermediate. In a subsequent report of deracemization of primary allene amides 68 from the same group^36^ (Scheme 11b), mechanistic studies and DFT calculations were performed to verify that the binding behavior of the substrate and the catalyst was crucial for the enantioselectivity. The structure of the triplet intermediates from 68 was also characterized. Continuing with their interest,^37^ the Bach group applied their strategy in the deracemization of chiral allenes 70–73 containing five-membered lactam moieties and chiral tetra-substituted alkenes.

## 5. Conclusions and outlook

Recent years have witnessed considerable efforts in the development of photocatalytic synthesis of structurally diversified axially chiral compounds. The stereocontrol of radical intermediates generated from photocatalyzed SET has been successfully developed, moreover, simultaneous control of axial and central chirality has been achieved. Relying on the photoinduced generation of key intermediates from simple starting materials, the asymmetric relay catalytic system of photocatalysis and organocatalysis has also been developed. A breakthrough in deracemization reactions relying on photocatalyzed energy transfer has been successfully explored. These general and sustainable methods greatly expand the scope and functional diversity of axially chiral compounds, opening new avenues in asymmetric catalysis. Despite the current impressive advancements, this field is still a burgeoning research area and some challenging tasks need to be addressed. The scope of radical precursors and the catalytic modes are currently limited and worth of further exploration. In deracemization reactions, the substrates are limited to lactams, indicating a need for the development of more stereo-induced modes. Moreover, synthesizing other types of axially chiral compounds beyond biaryls, styrenes and allenes is highly desirable. The construction of atropisomeric multi-axis systems and the incorporation of other chiral elements, such as central and planar chirality, will be of great interest. Given the significant achievements made so far, we expect this research area will continue to grow and eventually be established as a robust catalytic platform in organic synthesis.

## Data availability

This is a review article, and our manuscript does not contain any new data.

## Author contributions

Z. Zhang and L. Dai conceived and collaboratively drafted the manuscript.

## Conflicts of interest

There are no conflicts to declare.
